# Supplementary material for: Copulation Duration and Sperm Precedence with Reference to Larval Diapause Induction in Monochamus alternatus Hope (Coleoptera: Cerambycidae)
Source: Insects. 2024 Apr 8;15(4):255. doi: 10.3390/insects15040255 (PMC11050047; doi:10.3390/insects15040255)
Supplement: Supplementary file 1 [file insects-15-00255-s001.zip › Table_S1.zip!/Table_S1.pdf]

Table S1. Rearing conditions and food of *Monochamus alternatus alternatus* and *M. a. endai* populations used in Experiments 1—3.

**Experiment**	**1**	**2 and 3**	
**Insect**	***M. a. endai***	***M. a. alternatus***	***M. a. endai***
**Rearing conditions of immature stages** [1]	25°C, LD 12:12 h	25°C, LD 16:8 h	25°C, LD 16:8 h
**Larval food** [2]	*P. densiflora* bolts	*P. densiflora* and *P. thunbergii* bolts	*P. densiflora* bolts
**Conditions to terminate diapause** [1]	10°C, LD 12:12 h	N/A	10 °C, LD 8:16 h
**Term to terminate diapause**	5 months or longer	N/A	9.6 months
**Rearing conditions of adults** [1]	25°C, LD 12:12 h	25°C, LD 16:8 h	25°C, LD 16:8 h
**Adult food** [2]	*P. densiflora* twig	*P. densiflora* and *P. thunbergii* twig	*P. densiflora* and *P. thunbergii* twig
**Oviposition substrate** [2]	*P. densiflora* branch	*P. densiflora* and *P. thunbergii* branch	*P. densiflora* and *P. thunbergii* branch

[1] For example, LD 12:12 h represents a 12-h light/dark photoperiod.

[2] *P. densiflora* and *P. thunbergii* are *Pinus densiflora* and *Pinus thunbergii*, respectively.
