# Supplementary material for: Copulation Duration and Sperm Precedence with Reference to Larval Diapause Induction in Monochamus alternatus Hope (Coleoptera: Cerambycidae)
Source: Insects. 2024 Apr 8;15(4):255. doi: 10.3390/insects15040255 (PMC11050047; doi:10.3390/insects15040255)
Supplement: Supplementary file 1 [file insects-15-00255-s001.zip › Table_S2.zip!/Table_S2.pdf]

Table S2. Dimensions of *Pinus densiflora* bolts used to rear offspring of *Monochamus alternatus alternatus* (T) and *M. a. endai* (J) female adults that had experienced different types of mating in Experiments 2 and 3 in order to assess the second male sperm precedence (P_2 value).

Experiment	Type of mating (female × male)	Pine bolts used to rear offspring			
		Number	Length (cm)	Diameter (cm)	Bark surface area (cm^2)
2	**T × T**	40	15.0 ± 0.1	5.90 ± 0.22	277.6 ± 10.5
	**T × J after mating with a T male** [1]	40	15.0 ± 0.1	5.87 ± 0.23	277.1 ± 10.8
	**T × J after mating with a T male** [2]	37	15.0 ± 0.1	5.48 ± 0.22	258.5 ± 10.8
	**T × J**	36	15.1 ± 0.1	5.90 ± 0.25	279.6 ± 12.0
	**J × J**	36	15.0 ± 0.1	5.89 ± 0.24	277.7 ± 11.2
	**Total**	189	15.0 ± 0.0	5.81 ± 0.10	274.2 ± 4.9
	**Bartlett's test**				
	**χ^2 value (df =4)**		3.02	0.41	0.41
	***p* value**		0.554	0.982	0.982
	**One-way ANOVA**				
	***F* value (df = 4, 184)**		0.27	0.62	0.62
	***p* value**		0.896	0.649	0.649
3	**T × T**	31	15.0 ± 0.1	4.94 ± 0.47	231.9 ± 22.1
	**T × J after mating with a T male** [1]	31	14.9 ± 0.1	4.94 ± 0.47	230.1 ± 21.7
	**T × J**	31	15.0 ± 0.1	4.89 ± 0.46	231.3 ± 22.0
	**J × J**	31	15.0 ± 0.1	4.79 ± 0.43	224.4 ± 20.0
	**Total**	124	15.0 ± 0.0	4.89 ± 0.23	229.4 ± 10.7

**Bartlett's test**			
**χ^2 value (df =3)**	3.62	0.36	0.36
***p* value**	0.306	0.948	0.948
**One-way ANOVA**			
***F* value (df = 3, 120)**	0.96	0.02	0.03
***p* value**	0.416	0.996	0.995

[1] Pine bolts were used to rear offspring produced by T females 1—7 or 1—8 days after remating with a J male.

[2] Pine bolts were used to rear offspring produced by T females 20—37 days after remating with a J male.
